# Supplementary material for: MAIT cell clonal expansion and TCR repertoire shaping in human volunteers challenged with Salmonella Paratyphi A
Source: Nat Commun. 2018 Jan 17;9:253. doi: 10.1038/s41467-017-02540-x (PMC5772558; doi:10.1038/s41467-017-02540-x)
Supplement: Supplementary file 1 — Supplementary Information [file 41467_2017_2540_MOESM1_ESM.pdf]

Supplementary Fig. 1 - MAIT cell gating strategy for flow cytometric analysis and cell sorting

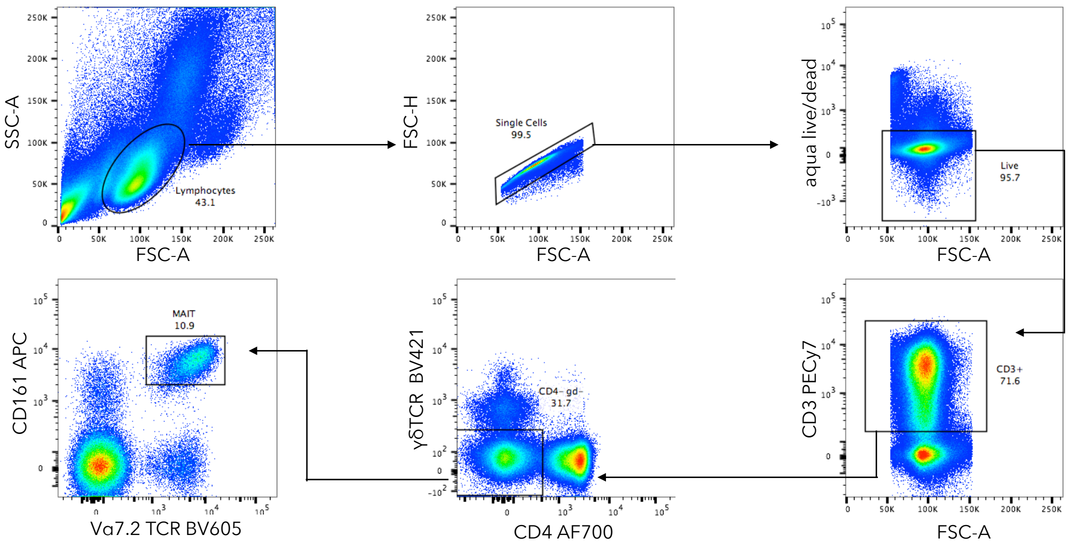

Flow cytometry and cell sorting gating strategy used for identifying MAIT cells in PBMCs. Cells were gated based on lymphocyte size using forward scatter (FSC) and side scatter (SSC) then gated on: single cells, live cells (live/dead aqua$^-$), CD3$^+$, γδ$^-$, CD4$^-$, CD161$^+$ and Vα7.2$^+$.

Supplementary Fig. 2 - MR1 tetramer staining of diagnosed donors' MAIT cells before, during and after infection

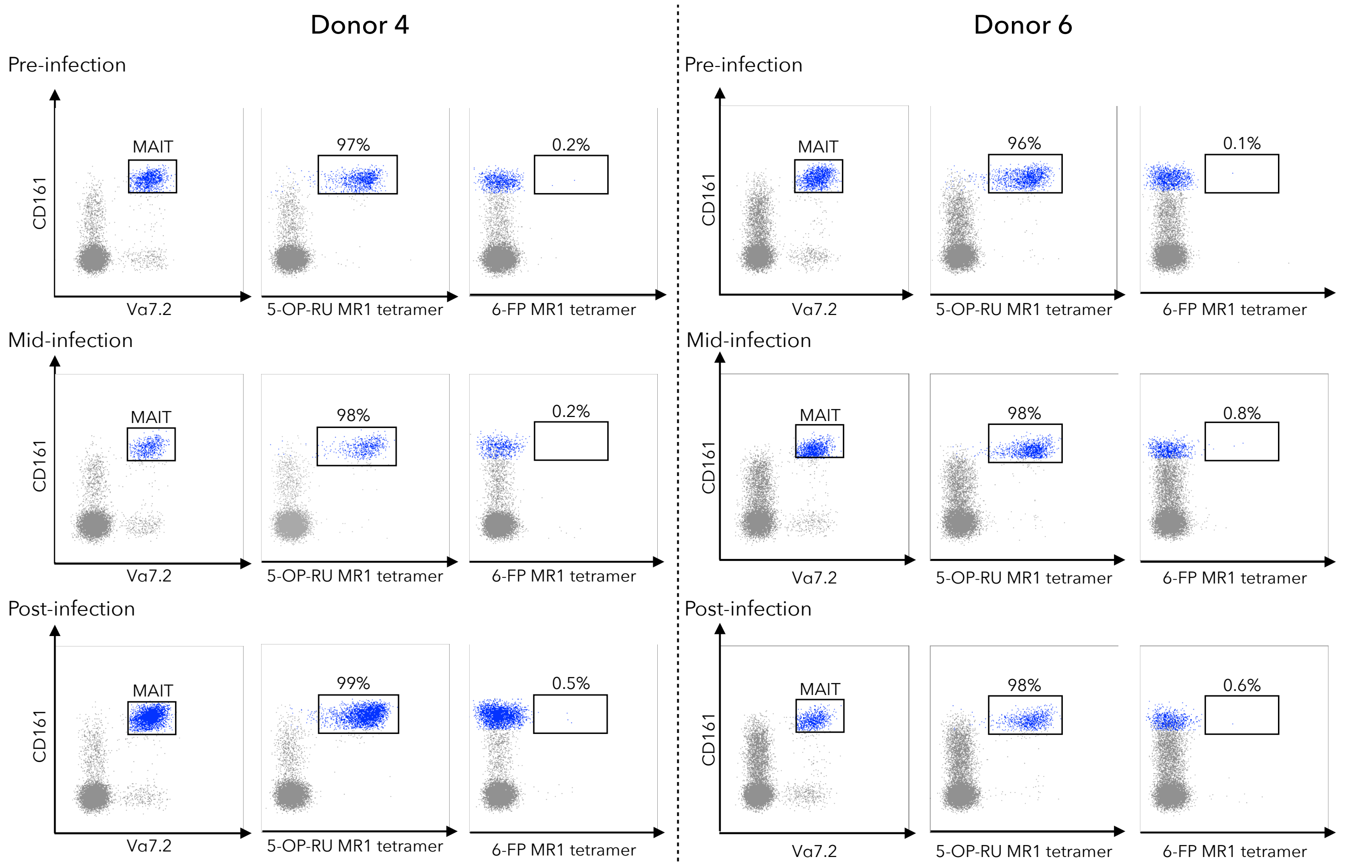

Frozen PBMCs from two diagnosed individuals taken at timepoints: day 0 (pre-infection), day 5–10 (96 hours post-diagnosis, mid-infection) and day 28 (post-infection) were thawed and analyzed by flow cytometry. Cells were gated on $CD3^{+}$ $\gamma\delta TCR^{-}$ $CD4^{-}$ as outlined in Supplementary Fig. 1. Plots on the left show $V\alpha7.2^{+}$ $CD161^{+}$ MAIT cells represented in blue. In the centre, plots show 5-OP-RU MR1 tetramer staining with $V\alpha7.2^{+}$ $CD161^{+}$ gated MAIT cells in blue and the percentage of tetramer positive gated $V\alpha7.2^{+}$ $CD161^{+}$ MAIT cells displayed. On the right, plots show control 6-FP MR1 tetramer staining with $V\alpha7.2^{+}$ $CD161^{+}$ MAIT cells in blue.

Supplementary Fig. 3 - Expression of proliferation, activation and chemokine receptors by MAIT cells at the peak of infection in diagnosed individuals

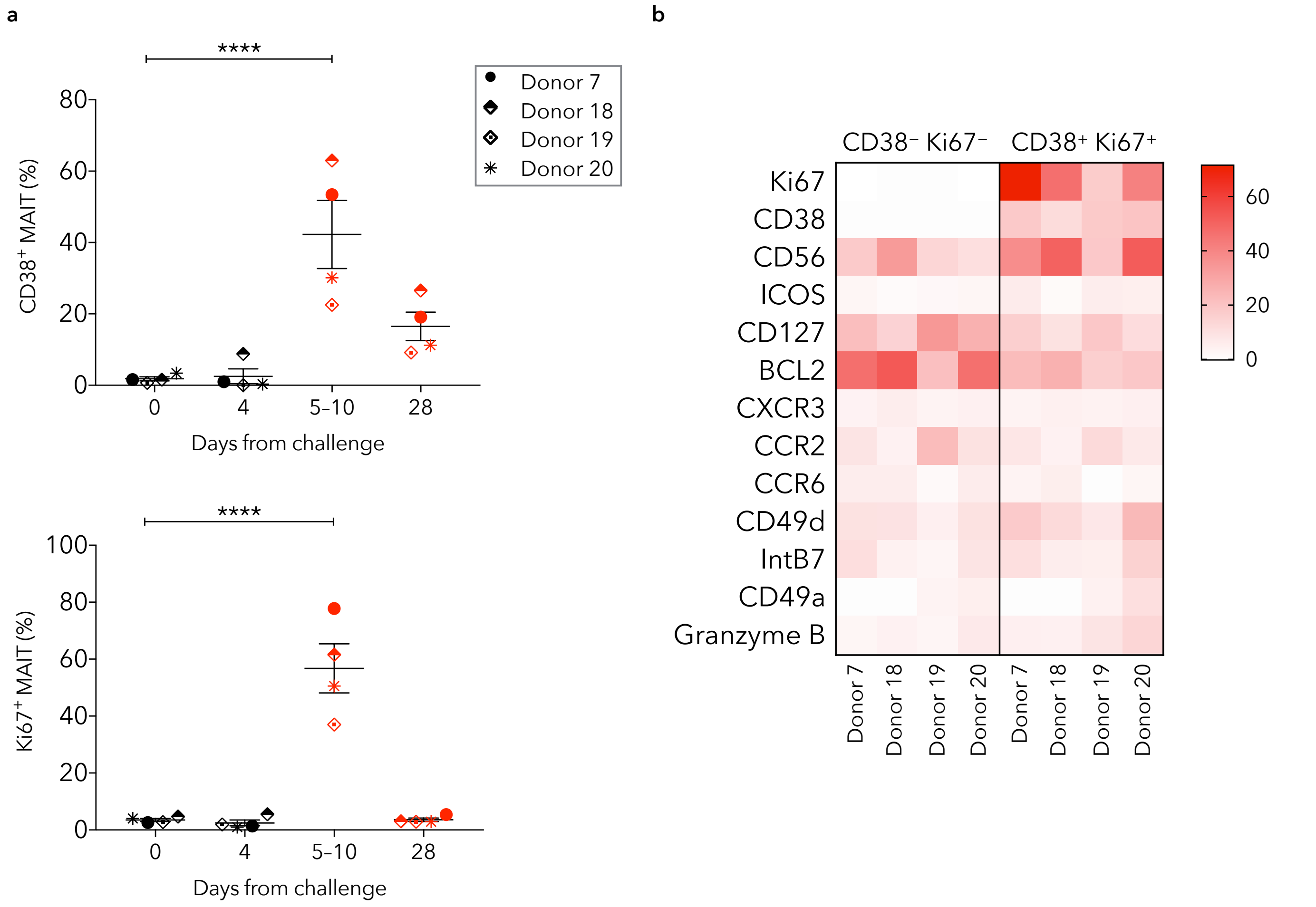

Frozen PBMCs from four diagnosed individuals taken at timepoints: day 0, day 4, day 5–10 (96 hours post-diagnosis) and day 28 were thawed and analyzed by mass cytometry. (a) Graphs show the expression of CD38 and Ki67 on MAIT cells over time. Numbers on x-axis represent days from bacterial challenge. Black symbols represent samples taken before diagnosis and red symbols represent samples taken on/after diagnosis with enteric fever. Line is at the mean and error bars represent standard error of the mean (SEM). (b) Heatmap shows the signal intensity for various activation and cell migration markers expressed by proliferating and activated MAIT cells (Ki67^{+} CD38^{+}) compared to the non-activated and non-proliferating (Ki67^{-} CD38^{-}) MAIT cells at the peak of infection (Day 5–10). One-way repeated measures ANOVA with Dunnett's test was used to assess statistical significance, where **** $P < 0.0001$.

Supplementary Fig. 4 - CyTOF validation of the expression of Ki67 and CD38 by diagnosed donors' MAIT cells using flow cytometry

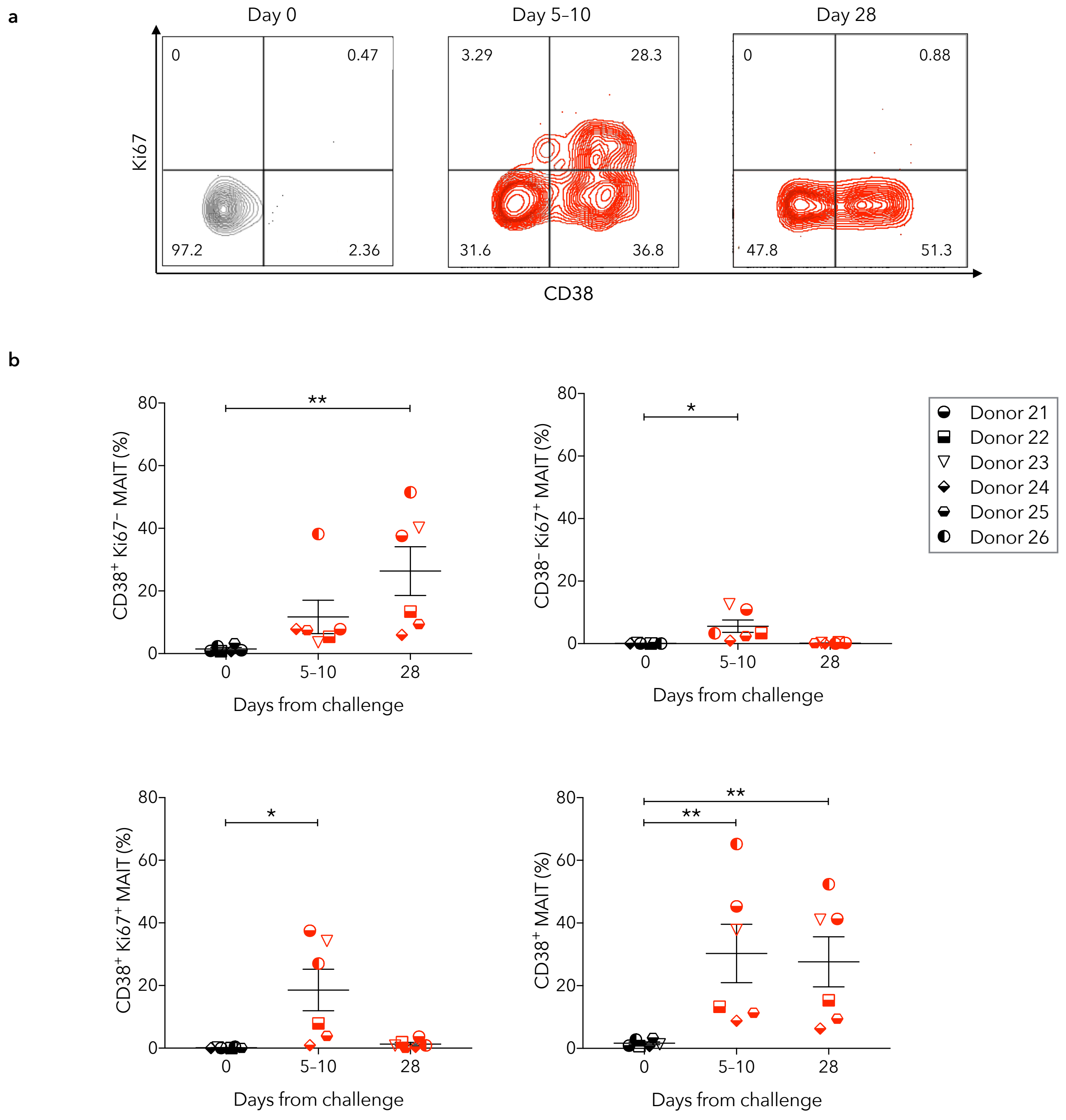

Fix/lysed whole blood from six diagnosed individuals at timepoints: day 0, day 5–10 (96 hours post-diagnosis) and day 28 were thawed and analyzed by flow cytometry. (a) Plots show an example of the expression of Ki67 and CD38 on MAIT cells from donor 26 over time. (b) Graphs summarize CD38 and Ki67 on MAIT cells from six donors. Numbers on x-axis represent days from bacterial challenge. Black symbols represent samples taken before diagnosis and red symbols represent samples taken on/after diagnosis with enteric fever. Line is at the mean and error bars represent SEM. One-way repeated measures ANOVA with Dunnett's test was used to assess statistical significance, where * $P < 0.05$, ** $P < 0.01$.

Supplementary Fig. 5 - Healthy MAIT cell response to *S.* Paratyphi A *in vitro* infection

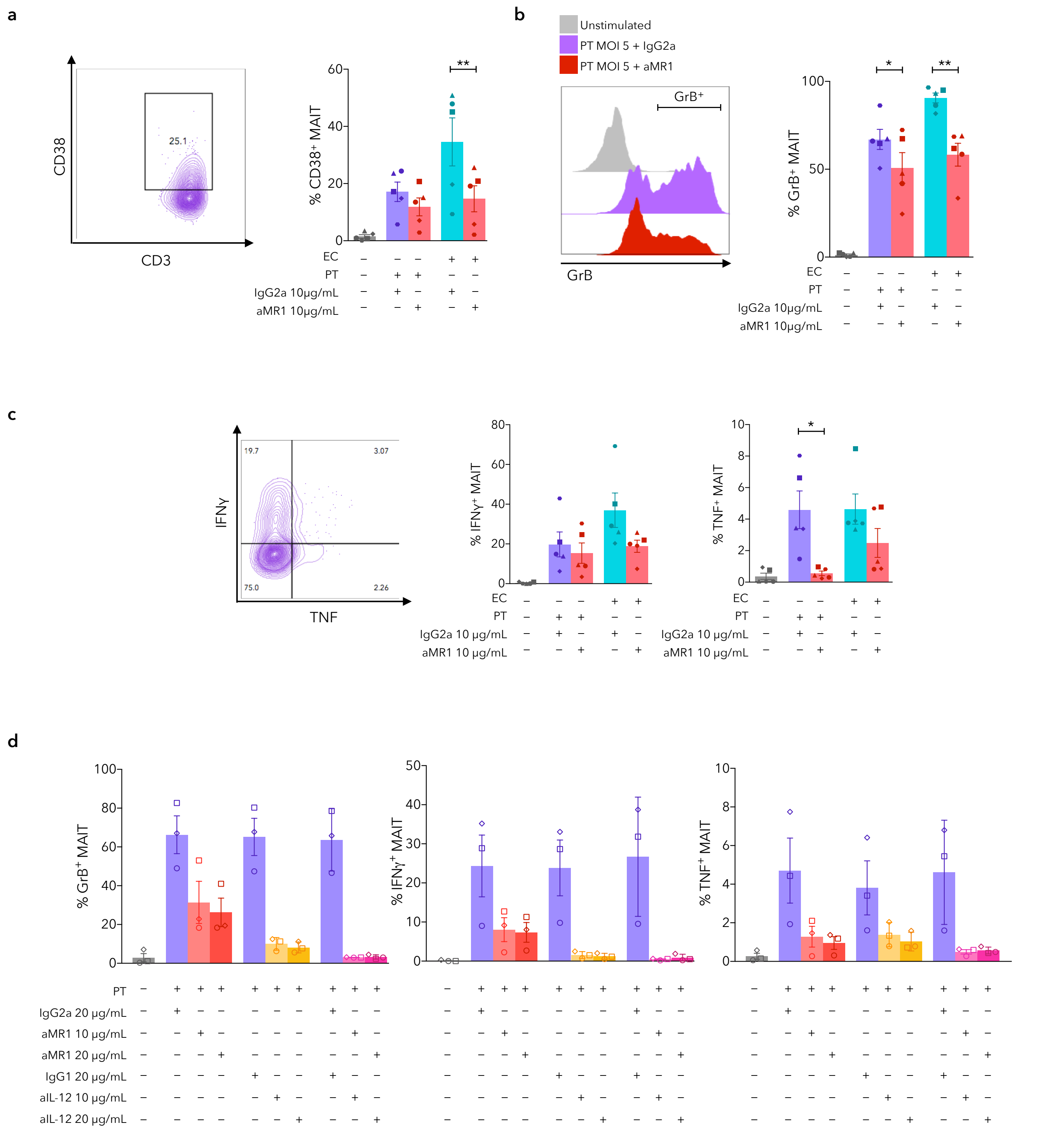

PBMCs were isolated from healthy donors and exposed to live *Salmonella enterica* serovar Paratyphi A (PT) multiplicity of infection (MOI) 5 or *Escherichia coli* (EC) MOI 5 *in vitro* with MR1 blocking antibody (aMR1) (n = 5). IgG2a isotype controls were used. Each symbol represents a donor. Cells were analyzed by flow cytometry and MAIT cells were assessed for expression of (a) CD38, (b) Granzyme B (GrB), and cytokines (c) IFNγ and TNF. Statistical significance was calculated using Student's two-tailed paired T test, where * $P < 0.05$ and ** $P < 0.01$. (d) Comprehensive blocking experiments of the effector function of MAIT cells from healthy donors (n = 3) in response to live *S.* Paratyphi A MOI 5 using varying concentrations of aMR1 and IL-12p40 blocking antibody with corresponding isotype controls IgG2a and IgG1, respectively.

Supplementary Fig. 6 - TCRβ chain usage in healthy individuals over time

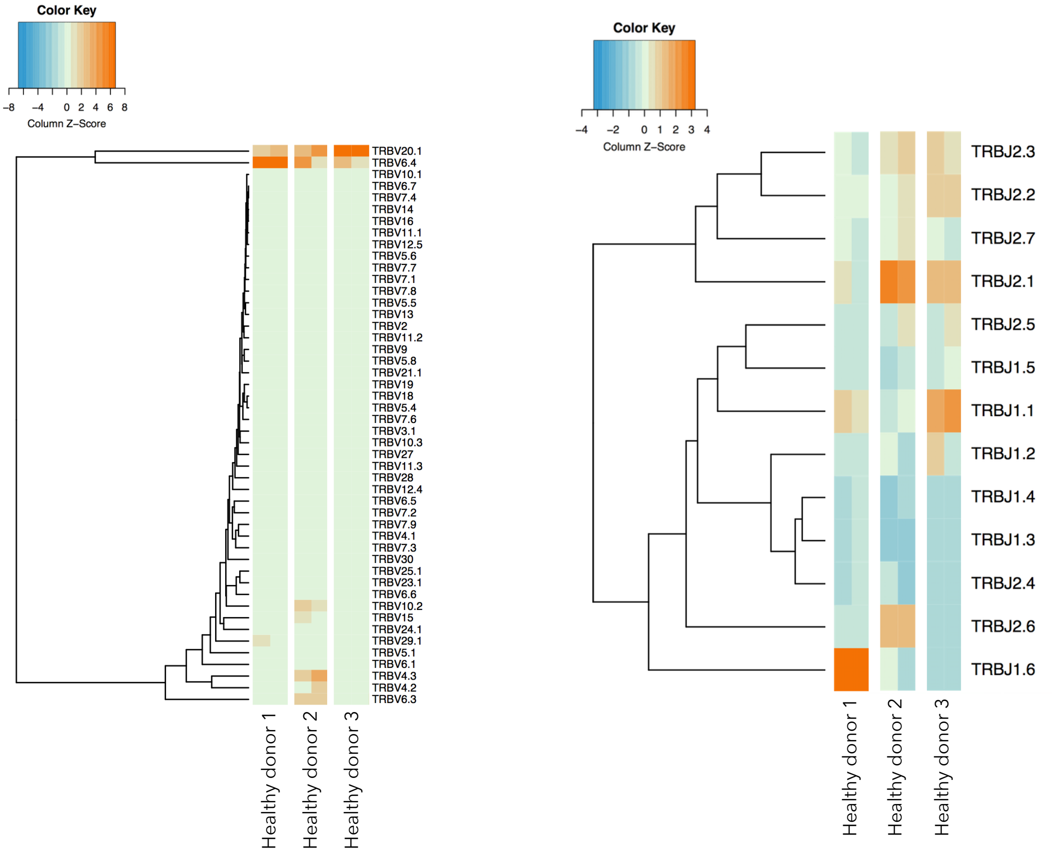

Frozen PBMCs collected 21 days apart from three healthy control donors were sorted for MAIT cells and then analyzed for TCRβ chain usage. The weighted TRBV and TRBJ usage profiles are shown as heatmaps with hierarchical clustering performed using euclidean distance. Bottom x-axis labels indicate healthy donor number.

Supplementary Fig. 7 - Occupied homeostatic space from individuals not diagnosed with infection

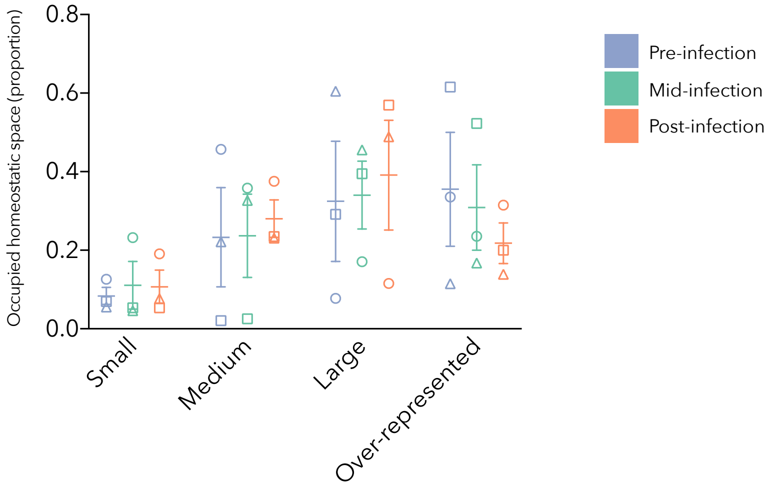

Frozen PBMCs from three individuals challenged with *S.* Paratyphi A and not diagnosed with enteric fever were sorted for MAIT cells (before, during and after infection) and the TCR repertoire analyzed. Graph shows summary of occupied homeostatic space taken up by MAIT cell clonotypes in not diagnosed individuals. Measured as a proportion taken up by over-represented (0.05–1), large (0.01–0.05), medium (0.001–0.01), and small clonotypes (0–0.001). Each symbol represents a volunteer.

Supplementary Fig. 8 - Diagnosed individuals repertoire overlap and top five clonotypes pre- vs. post-infection

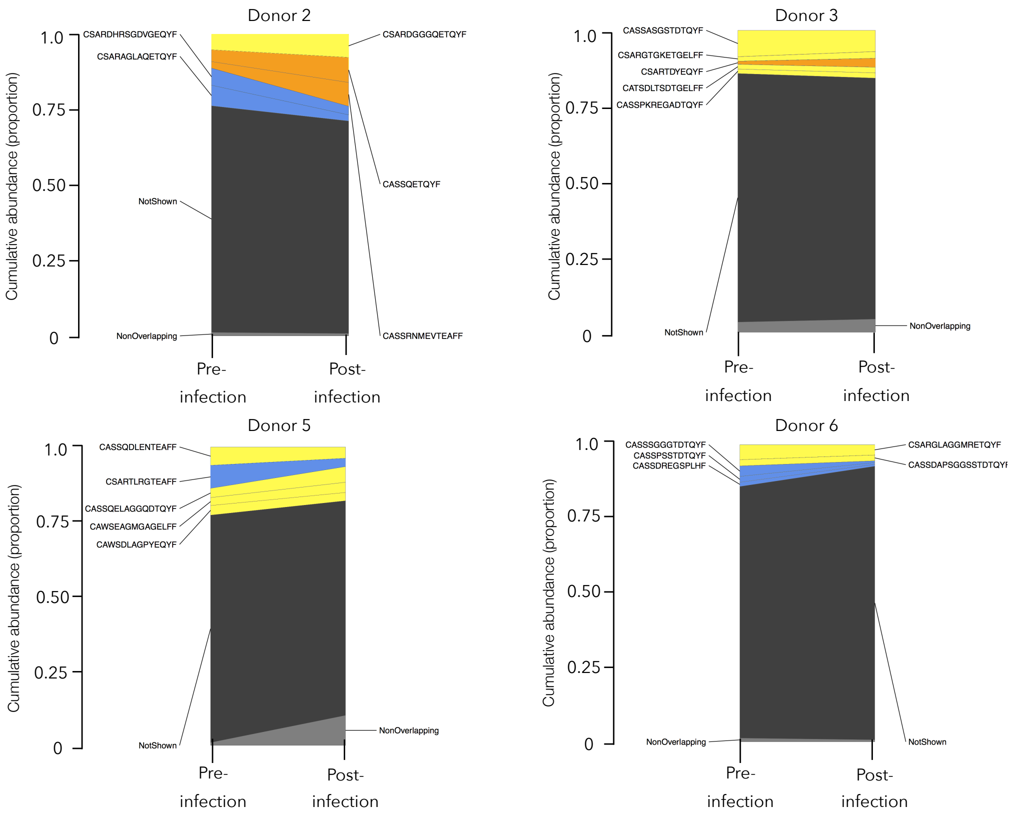

Frozen PBMCs from individuals challenged with *S.* Paratyphi A and diagnosed with enteric fever were sorted for MAIT cells (before and after infection) and the TCR repertoire analyzed. Repertoire overlap plots show cumulative abundance of clonotypes before and after infection in diagnosed donors: 2, 3, 5 and 6. The top five clonotypes between the two time points are shown. Orange indicates expanded clone (>2-fold increase), blue indicates contracted clone (>2-fold decrease) and yellow indicates unchanged.

Supplementary Fig. 9 - Diagnosed individual TRAJ usage during infection

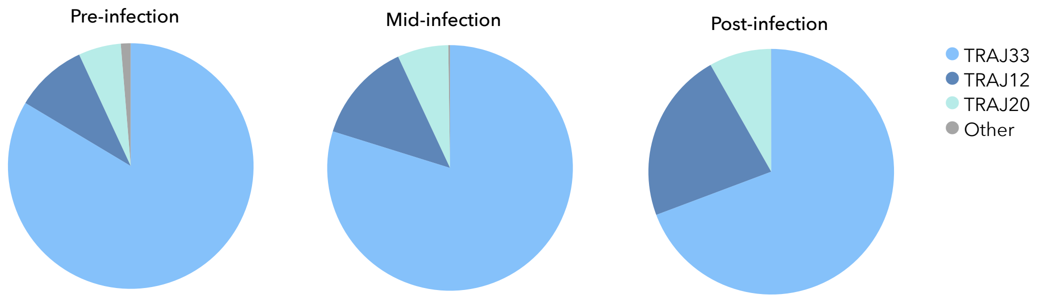

Frozen PBMCs from donor 1 challenged with *S.* Paratyphi A and diagnosed with enteric fever were sorted for MAIT cells (before, during and after infection) and the TCRα chain usage analyzed. Pie charts show the proportion of TRAV1-2 TCRα chains using different TRAJ regions.

Supplementary Fig. 10 - Jurkat.MAIT gating strategy for flow cytometry

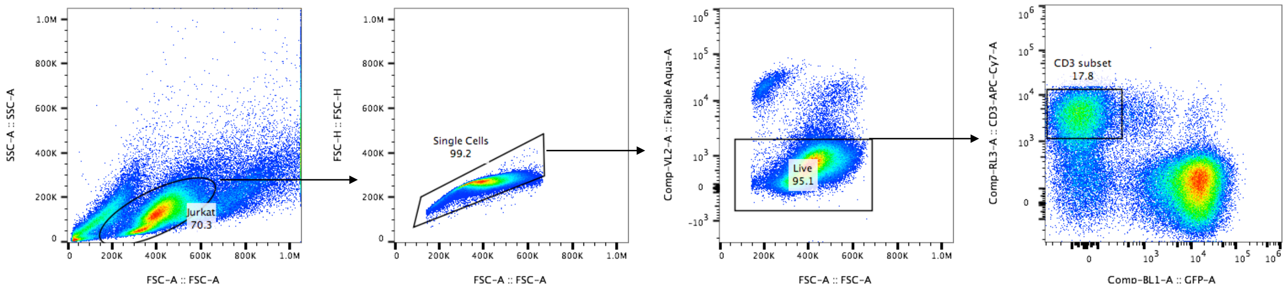

Jurkat.MAIT cells were made by transducing the MAIT TCRα chain into a vector that contained a TCRβ chain from either an expanded or contracted clonotype from donor 1 or donor 3. Lines were stained with CD3 and gated on the $CD3^{+}$ and GFP^{-} (where appropriate, to gate out GFP^{+} C1R.MR1 cells).

Supplementary Fig. 11 - Vα7.2 expression and CD3 overlay for Jurkat.MAIT lines

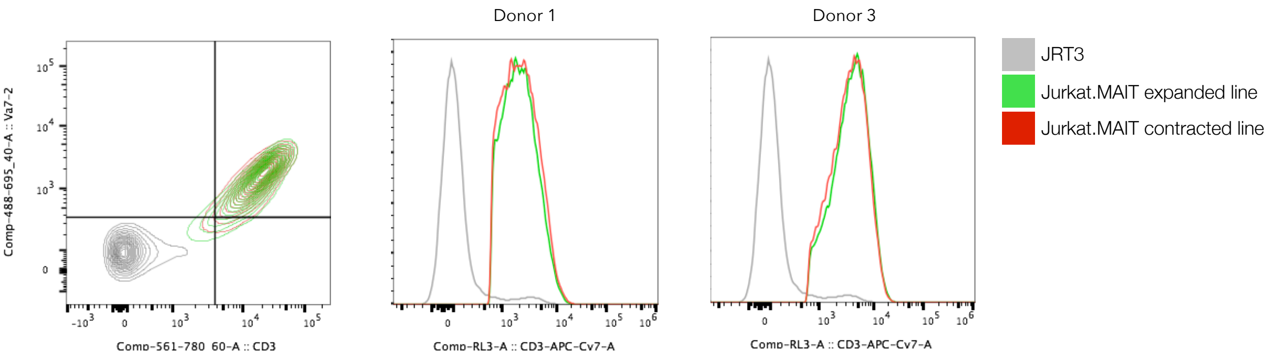

Jurkat.MAIT cells were made by transducing the MAIT cell TCRα chain into a vector that contained a TCRβ chain from either an expanded or contracted clonotype from donor 1 or donor 3. For all functional analysis, the lines were stained with CD3 and gated on the $CD3^{+}$ GFP^{-} population. Contour plot shows expression of Vα7.2, confirming TCRβ chain pairing with MAIT cell TCRα chain. Histogram shows the $CD3^{+}$ gate overlay between the two lines compared to the untransduced JRT3 cell line.

Supplementary Fig. 12 - Dilution curve and MR1 blocking of Jurkat.MAIT lines CD69 expression in response to bacterial stimulation

a

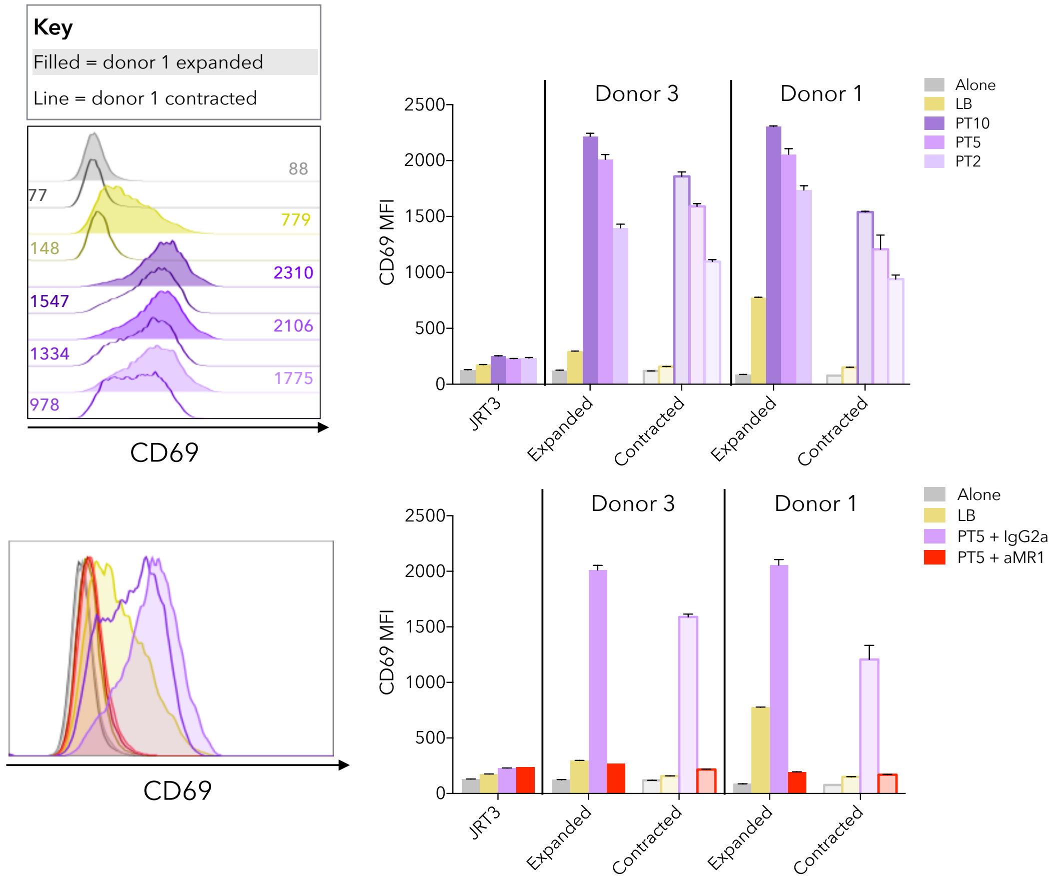

b

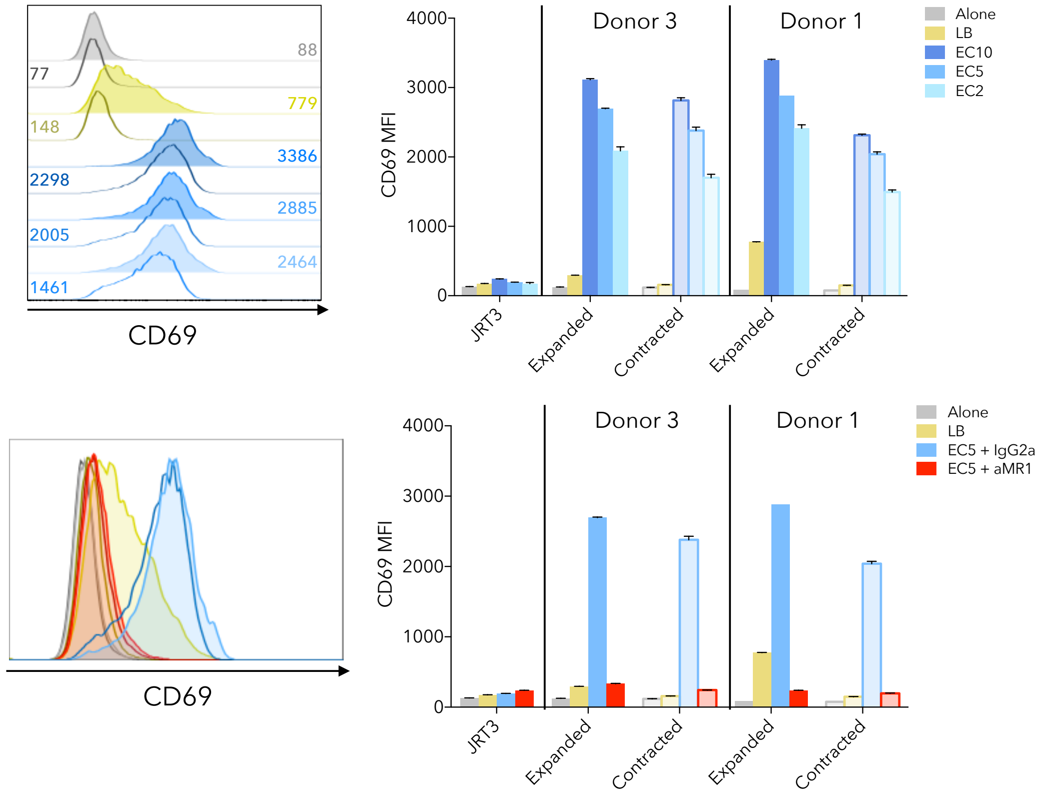

Jurkat.MAIT cells were made by transducing the MAIT TCRα chain into a vector that contained a TCRβ chain from either an expanded or contracted clonotype from donor 1 and donor 3 post-infection. Histograms show the CD69 expression from the $CD3^{+}$ population of donor 1 expanded (filled histogram) or contracted (line/no fill histogram). The dilution curve histograms show the MFI value for the expanded (right axis) or contracted (left axis) lines from donor 1. The overlay histograms shows CD69 expression with either isotype control (IgG2a) or MR1 blocking antibody (red) for donor 1. Graphs summarize the results for (a) *S.* Paratyphi A (PT) supernatant, (b) *E. coli* (EC) supernatant with number indicating volume of supernatant (μL) added. Samples were run in technical duplicates and data is representative of at least two independent experiments. LB; L-Broth.

Supplementary Fig. 13 - Dilution curve and MR1 blocking of Jurkat.MAIT lines CD69 expression in response to ligand stimulation

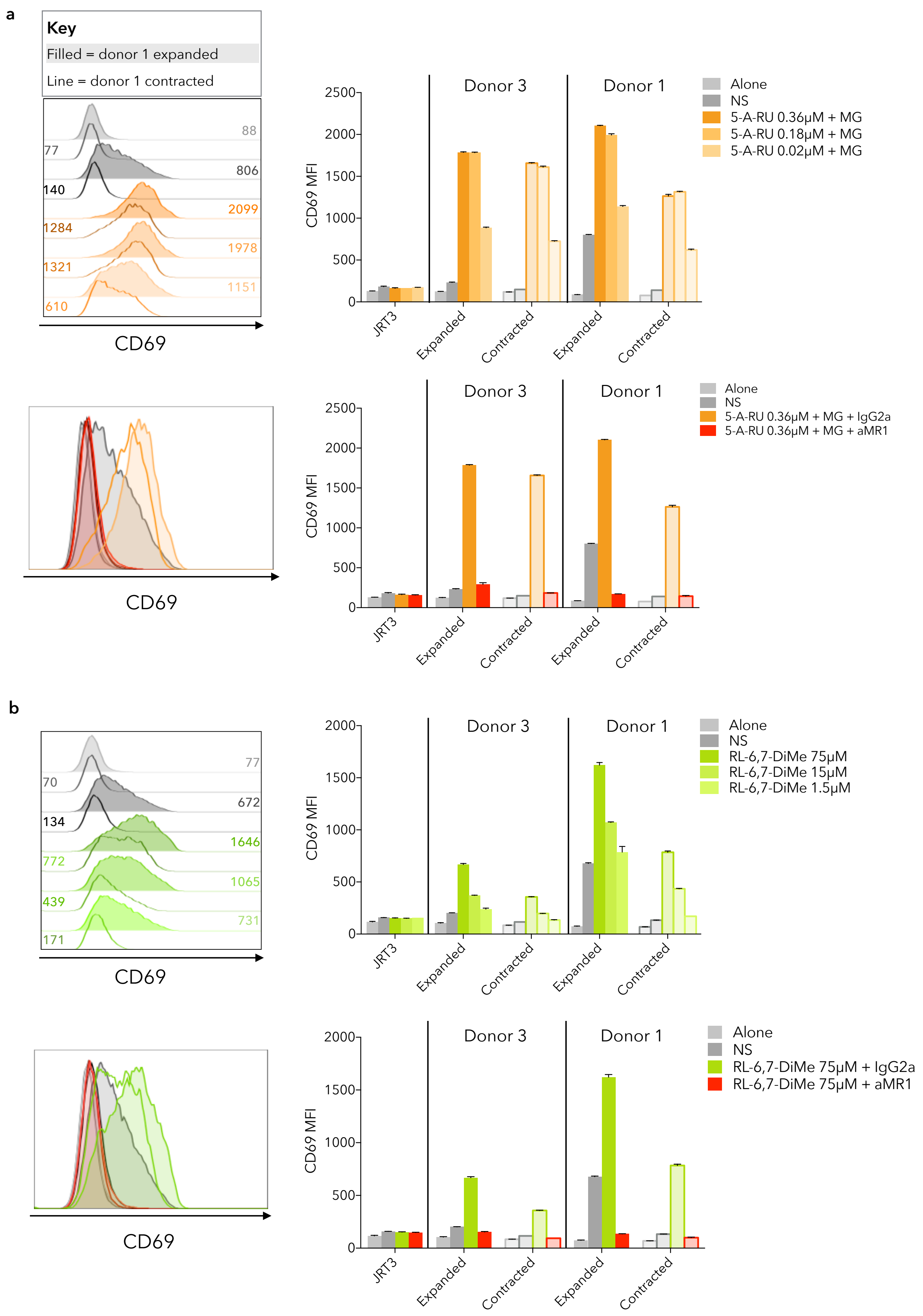

Jurkat.MAIT cells were made by transducing the MAIT TCRα chain into a vector that contained a TCRβ chain from either an expanded or contracted clonotype from donor 1 and donor 3 post-infection. Histograms show the CD69 expression from the $CD3^{+}$ population of donor 1 expanded (filled histogram) or contracted (line/no fill histogram). The dilution curve histograms show the MFI value for the expanded (right axis) or contracted (left axis) lines from donor 1. The overlay histograms shows CD69 expression with either isotype control (IgG2a) or MR1 blocking antibody (red) for donor 1. Graphs summarize the results for (a) 5-A-RU with 10 μM methylglyoxal (MG) and (B) 6,7-dimethylribityl lumazine (RL-6,7-DiMe). Samples were run in technical duplicates and data is representative of at least two independent experiments. NS; no stimulation.

Supplementary Fig. 14 - 5-OP-RU MR1 tetramer decay on Jurkat.MAIT lines

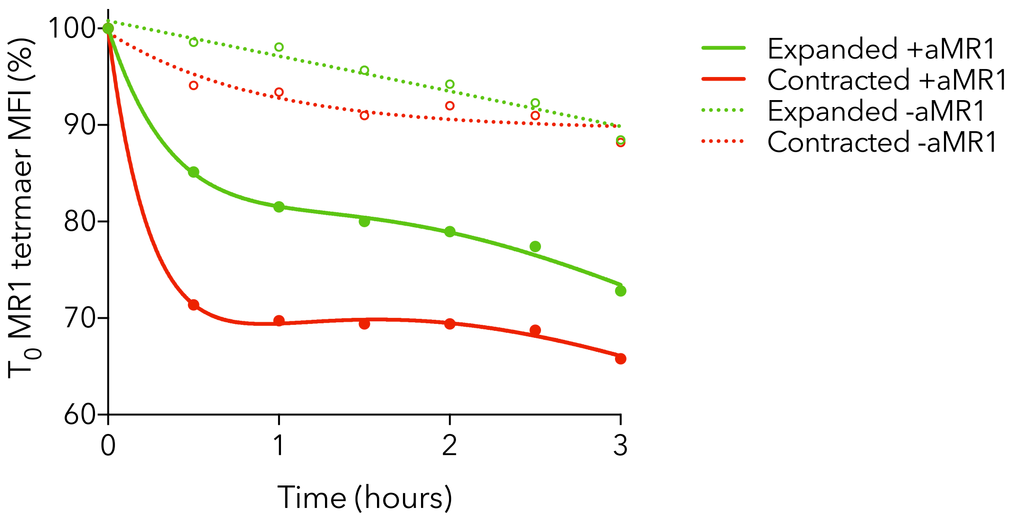

Jurkat.MAIT cells were made by transducing the MAIT TCRα chain into a vector that contained a TCRβ chain from either an expanded (green) or contracted (red) clonotype from donor 1. Line graph shows the staining intensity as a percentage of staining at Time = 0 (T_0) of the 5-OP-RU MR1 tetramer over time when cells are incubated at 37°C. The tetramer decay with and without the presence of 20 µg/mL MR1 blocking antibody (aMR1) is plotted with an exponential decay equation model fitted.

Supplementary Fig. 15 - Effect of dialyzed FBS on activation of peptide-specific T cells

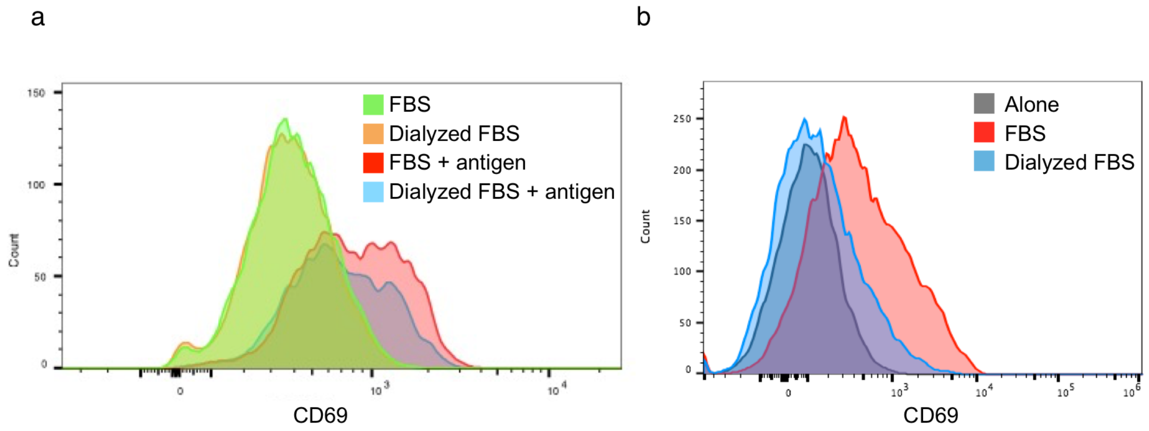

(a) CD4+ T cells specific for the *S. enterica* protein CdtB were stimulated with 3 µg/mL CdtB peptide for 18 hours in culture media containing either normal or dialyzed FBS. Plot shows their expression of CD69. (b) Plot shows the expression of CD69 on the expanded Jurkat.MAIT line from donor 1 in the presence of normal versus dialyzed FBS.

Supplementary Table 1 – Mass cytometry primary surface antibody cocktail

ANTIBODY	CONJUGATION	CLONE	COMPANY
Vδ1	FITC	REA173	Miltenyi Biotec
TCRγδ	PE	5A6.E9	Invitrogen

Supplementary Table 2 – Mass cytometry secondary and primary metal-conjugated antibody cocktail

ANTIBODY	METAL	CLONE	COMPANY
**CD45**	89	HI30	Fluidigm
**Qdot800-CD14**	112/114	TüK4	Invitrogen
**CD57**	115	HCD57	Biolegend
**anti-PE**	139	PE001	Biolegend
**CD3**	140	UCHT1	Biolegend
**HLA-DR**	141	L243	Biolegend
**CLA**	142	HECA-452	Biolegend
**Vα7.2**	143	3C10	Biolegend
**CD45RA**	145	HI100	Biolegend
**CD8α**	146	SK1	Biolegend
**CD45RO**	147	UCHL1	Biolegend
**Vδ2**	148	B6	Biolegend
**CD4**	149	SK3	Biolegend
**CD103**	150	B-Ly7	eBioscience
**CCR4**	151	205410	R&D Systems
**CD49a**	154	TS2/7	Biolegend
**CCR7**	156	150503	R&D Systems
**CD27**	157	LG.7F9	eBioscience
**CD56**	158	NCAM16.2	BD Biosciences
**ICOS**	159	C398.4A	Biolegend
**PD-1**	160	eBioJ105	eBioscience
**anti-FITC**	161	FIT-22	Biolegend
**CD161**	162	HP-3G10	Biolegend
**CXCR3**	163	49801	R&D Systems
**CCR9**	164	L053E8	Biolegend
**CD38**	165	HIT2	Biolegend
**CXCR5**	166	RF8B2	BD Biosciences
**CD49d**	167	9F10	Biolegend
**CCR2**	168	K036C2	Biolegend
**CD25**	169	M-A251	Biolegend
**CCR6**	170	G034E3	Biolegend
**Integrin β7**	171	FIB504	Biolegend
**CD19**	173	HIB19	Biolegend
**CX3CR1**	174	K0124E1	Biolegend
**CCR5**	175	HEK/1/85a	Abcam
**CD127**	176	A019D5	Biolegend
**CD16**	209	3G8	Fluidigm

Supplementary Table 3 – Primary intracellular antibody cocktail

ANTIBODY	CONJUGATION	CLONE	COMPANY
**Ki-67**	152	B56	BD Biosciences
**Granzyme B**	144	CLB-GB11	Abcam
**Foxp3**	Biotin	PCH101	eBioscience
**BCL-2**	172	100	Biolegend
